# The Combined Photosensitizers in Antimicrobial Photodynamic Therapy: The Case of Methylene Blue and Photodithazine Against *Klebsiella pneumoniae*

**DOI:** 10.3390/ijms262010211

**Published:** 2025-10-21

**Authors:** Koteswara Rao Yerra, Jennifer M. Soares, Vanderlei S. Bagnato

**Affiliations:** 1 Department of Biomedical Engineering, College of Engineering, Texas A&M University, College Station, TX 77843, USA; 2 São Carlos Institute of Physics, University of São Paulo, São Carlos 13566-590, SP, Brazil

**Keywords:** *Klebsiella pneumoniae*, photosensitizers, simultaneous treatment, sequential treatment, Photobleaching

## Abstract

Photodynamic therapy (PDT) is a promising antimicrobial strategy whose efficacy depends largely on the photosensitizers (PSs) used. While conventional PDT relies on a single PS, recent studies suggest that combining different PSs may improve outcomes by introducing complementary mechanisms. However, such combinations also add complexity, as timing, composition, and PS interactions must be considered alongside bacterial structures, uptake pathways, and light dosimetry. This study investigated the effects of PSs, methylene blue (MB), Photodithazine (PDZ), and their combinations on the PDT of Gram-negative bacterium *Klebsiella pneumoniae*. MB-mediated PDT demonstrated greater antibacterial effectiveness than PDZ-PDT. The combination of MB and PDZ produced varying results. When applied simultaneously, PDZ dose-dependently decreased MB’s antibacterial activity. Sequential treatment with PDZ followed by MB showed only slight antagonism compared to MB alone, while the reverse order (MB → PDZ) nearly abolished MB’s activity. Since both PSs are activated at the same wavelength (660 nm), their combined use was not additive. Photobleaching was performed on individuals and combined PSs to compare inactivation results with changes in chemical properties under red light (660 nm). This study highlights the limitations of using two photosensitizers together in antimicrobial photodynamic therapy and emphasizes the need for further optimization of combination protocols.

## 1. Introduction

Pathogenic bacteria, especially multidrug-resistant (MDR) strains, present a significant global health threat. Unrestricted antibiotic use in medicine and animal farming has hastened antimicrobial resistance (AR), diminishing the effectiveness of current treatments [1]. Over time, the emergence of truly new antibiotic classes has slowed, while AR rates have continued to rise. Most bacterial infections are caused by MDR strains such as *Enterococcus faecium*, *Staphylococcus aureus*, *Klebsiella pneumoniae*, *Acinetobacter baumannii*, *Pseudomonas aeruginosa*, and *Enterobacter* spp. (ESKAPE) [2]. Many of these strains form biofilms, which further enhance antibiotic resistance [2]. A CDC report from July 2024, Antimicrobial Resistance Threats in the United States (2021–2022), noted a 20% increase in hospital-acquired infections caused by ESKAPE species following the COVID-19 pandemic (2019). There is an urgent need for effective alternative therapies for MDR pathogens. Recent research indicates that antimicrobial photodynamic therapy (aPDT) is a promising alternative for combating pathogenic, resistant bacteria [3]. The mechanism of aPDT involves the uptake of a non-toxic dye called a photosensitizer (PS) by the target cell. Upon activation by specific wavelengths of light in the presence of oxygen, the PS generates cytotoxic reactive oxygen species (ROS), primarily singlet oxygen (^1^O_2_) and free radicals. These ROS broadly damage bacterial membranes, proteins, and nucleic acids, leading to irreversible cell damage resulting in cell death [4]. Compared to traditional antimicrobial treatments, aPDT offers several advantages, including the eradication of resistant bacteria, local delivery of PS, site-specific effects where both PS and light are applied simultaneously, and immediate action [4]. Due to the non-specific and multi-targeted nature of ROS-induced cell death and the brief exposure to PS, bacterial cells are unlikely to develop resistance to aPDT as protective factors are less likely to be expressed. Although local applications of aPDT avoid systemic side effects, it often requires high concentrations of PS and intense light doses to eliminate bacteria, which can potentially harm host cells and impair local immune responses [4]. Therefore, the effectiveness of PDT is influenced by several factors, including the chemical nature of the PS, its interaction with microbial cells, and the parameters of light exposure. Consequently, a significant volume of research has been dedicated to identifying and optimizing photosensitizing molecules to enhance therapeutic outcomes.

Recent advances in photosensitizer (PS) design include natural products (e.g., curcumin, riboflavin, etc.), nanostructures (e.g., fullerenes, titanium dioxide, etc.), synthetic dyes [e.g., methylene blue (MB), toluidine blue, etc.], and tetra-pyrrole structures (e.g., porphyrins, chlorins, etc.), each offering unique benefits in biocompatibility, ROS production, targeting ability, and tissue penetration. Natural products like melanin derivatives show good biocompatibility but are limited by poor photostability and weak absorption within the therapeutic window [5]. Noble metal complexes, including Ru (II), Ir (III), and Pt-based PSs, utilize strong spin–orbit coupling to enhance intersystem crossing and achieve high singlet oxygen (^1^O_2_) yields [6]. Carbon-based nanostructures such as graphene oxide and carbon dots have a broad absorption spectrum and easy surface functionalization for bacterial targeting [7]. Tetra-pyrrole compounds, often called the *pigments of life*, are some of the earliest known PSs and can absorb light at higher wavelengths [8]. Synthetic dyes like phthalocyanines and cyanines enable deeper tissue penetration and fluorescence-guided therapy [9]. Recently, earth-abundant iron-based coordination complexes have become sustainable, cost-effective options to precious metals, showing promising photochemical activity with less long-term toxicity [10].

Traditionally, photodynamic therapy (PDT) protocols employ a single photosensitizer (PS), with efficacy optimized by adjusting parameters such as incubation time and light dose. However, single-PS PDT remains constrained by several limitations, including oxygen dependence, restricted light penetration, and the potential risk of treatment resistance [11]. Consequently, the design and optimization of highly effective antimicrobial PDT (aPDT) remain a significant challenge, requiring continued fundamental research. Recent advances indicate that combining photosensitizers from different chemical classes may provide new opportunities to enhance photodynamic inactivation. Such combinations introduce additional variables—including PS composition, sequence and timing of administration, and inter-PS interactions—that can substantially influence therapeutic outcomes. Importantly, the presence of one PS may modulate the photophysical or biological activity of another, underscoring the complexity of designing effective PS mixtures [11,12]. Several studies highlight the therapeutic potential of this strategy. For instance, benzophenone combined with vitamins B1, B6, or K3 produced synergistic bactericidal effects, achieving up to a 4-log reduction in viable counts of *Escherichia coli*, *Bacillus cereus*, *Staphylococcus aureus*, and *Klebsiella pneumoniae* [13]. Likewise, co-application of hematoporphyrin derivative (HpD) and meso-tetrahydroxyphenylchlorin (mTHPC) demonstrated additive activity against *S. aureus* [14]. In contrast, not all PS pairings are advantageous; for example, hypericin was shown to promote bacterial growth and antagonize the effects of Photofrin II and mTHPC against *S. aureus* [15]. Collectively, these findings emphasize the need for rational design, and careful selection of the optimal PS mix for aPDT remains a considerable challenge.

**Figure 1:**
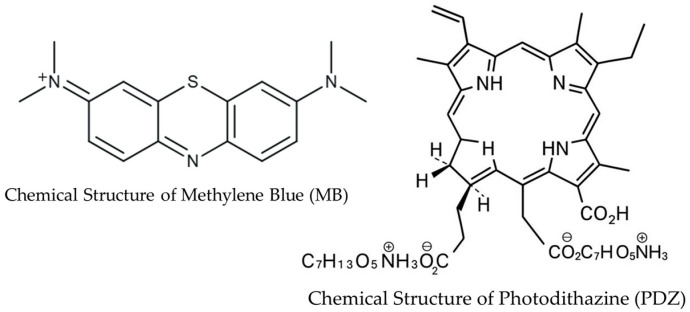
Chemical structures of methylene blue (MB) and Photodithazine (PDZ) [18].

The effectiveness of aPDT depends on multiple factors, including photosensitizer (PS) chemistry, bacterial envelope properties, uptake, photochemical pathways, and light dosimetry. These variables complicate treatment design and highlight the need for systematic strategies to optimize PS combinations. Here, we examine the cooperative and antagonistic interactions between two structurally distinct PSs—methylene blue (MB) and Photodithazine (PDZ)—against *Klebsiella pneumoniae*. MB, an FDA-approved phenothiazine dye, combines low toxicity with strong antibacterial activity, while PDZ, a cationic PS, demonstrates broad-spectrum activity, biofilm disruption, and efficacy against drug-resistant pathogens [16,17]. We selected MB and PDZ based on their well-established photodynamic properties, overlapping absorption in the red region (around 660 nm), and their documented efficacy in antimicrobial photodynamic therapy. These complementary characteristics make them suitable candidates for investigating potential synergistic or competitive effects in bacterial inactivation. The chemical structures of MB and PDZ are shown in Figure 1. To complement biological assays, we developed a photobleaching model capturing singlet oxygen (^1^O_2_) generation and PS degradation under irradiation. This integrated approach links mechanistic insights with antimicrobial outcomes, providing a framework for the rational design of next-generation aPDT strategies.

## 2. Results

### 2.1. Effect of Methylene Blue (MB) and Photodithazine (PDZ) Against Klebsiella pneumoniae

**Figure 2:**
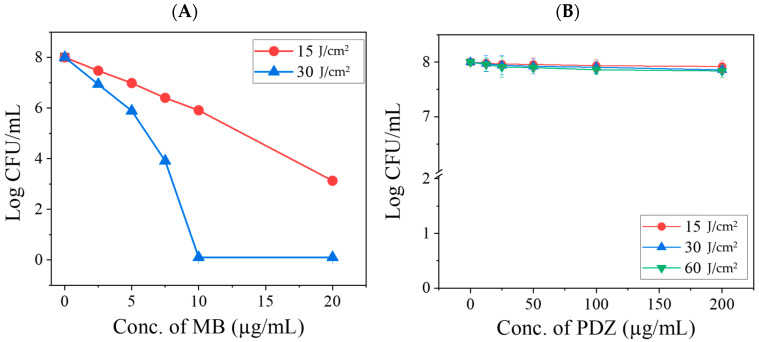
Evaluation of the photodynamic activity of each photosensitizer (PS), methylene blue (MB) (**A**), and Photodithazine (PDZ) (**B**), on *K. pneumoniae* irradiated with red LED light (660 nm). Results are presented as mean and standard deviation. Statistical significance was determined by comparing the treated group (PS + PDT) with the “only PS” control group at each concentration level.

The photodynamic activity of Methylene Blue (MB) and Photodithazine (PDZ) against *Klebsiella pneumoniae* was first evaluated individually across a range of concentrations and light doses. Both photosensitizers were excited at 660 nm with fluences from 15 to 60 J/cm^2^. Neither MB (≤20 µg/mL) nor PDZ (≤200 µg/mL) showed antibacterial activity in the absence of light. Likewise, irradiation up to 60 J/cm^2^ without photosensitizer did not affect bacterial growth; therefore, these control data are not included in Figure 2. When combined with light, MB demonstrated strong photodynamic activity in a concentration- and fluence-dependent manner (Figure 2). At an irradiation dose of 60 J/cm^2^ with MB, complete *K. pneumoniae* bacterial inhibition was consistently observed; therefore, the data were not included in Figure 2A, as the effect was complete bacterial inactivation. At MB concentrations of 2.5–20 µg/mL and a light dose of 15 J/cm^2^, *K. pneumoniae* viability was reduced by >0.5 to 4.9 log10 CFU/mL (Figure 2A). A concentration of 2.5 µg/mL MB with 30 J/cm^2^ irradiation resulted in a ~1.1 log10 reduction, whereas 10 µg/mL MB with 30 J/cm^2^ achieved complete bacterial inactivation (*p* < 0.05 vs. control). In contrast, PDZ exhibited only minimal activity under similar conditions. At the highest tested concentration (200 µg/mL) and 15 J/cm^2^ light dose, bacterial survival decreased by ~0.2 log10, with a maximum reduction of ~0.4 log10 observed at 60 J/cm^2^ (*p* < 0.05) (Figure 2B).

### 2.2. Combination Effect of PDZ and MB

**Figure 3:**
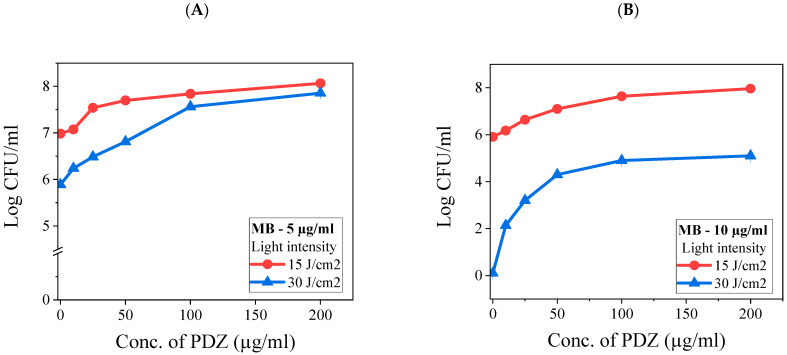
The combination effect of photosensitizers, methylene blue (MB) and Photodithazine (PDZ), on the viability of *Klebsiella pneumoniae*. A graph illustrates the relationship between the concentration of the photosensitizers (measured in µg/mL) and the resulting count of colony-forming units (CFU/mL). Specifically, panels (**A**) and (**B**) present data with MB concentrations held constant at 5 and 10 µg/mL, respectively, while varying the concentration of PDZ.

Figure 3 shows the photodynamic effects of simultaneous administration of MB and PDZ against *K. pneumoniae* planktonic cells. Photosensitizer concentrations were selected below their previously established maximum phototoxic doses to assess potential synergistic, additive, or antagonistic interactions. MB was tested at fixed concentrations of 5 µg/mL (Figure 3A) or 10 µg/mL (Figure 3B), while PDZ was varied from 12.5 to 200 µg/mL. These conditions were compared with the effects of each photosensitizer alone (Figure 2). PDZ concentrations ≤25 µg/mL produced minimal effects; however, higher concentrations progressively attenuated MB-mediated killing. At 15 J/cm^2^, MB (5 µg/mL) alone achieved a mean log10 reduction of 2.2, whereas PDZ (100 µg/mL) alone reduced viability by only 0.1 log10. When combined, the observed reduction was 0.2 log10, markedly below the expected additive effect of 2.3 log10, indicating 2.0 log10 of antagonism (Figure 3A). At PDZ concentrations of 100 and 200 µg/mL, MB’s photodynamic activity was completely abolished (Figure 3A).

**Figure 4:**
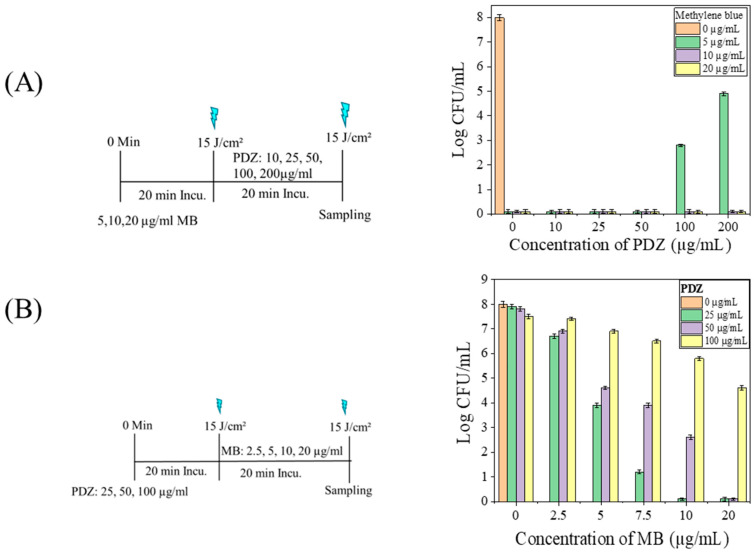
The sequential effect of photosensitizers, methylene blue (MB), and Photodithazine (PDZ), on the viability of *Klebsiella pneumoniae*. A graph illustrates the relationship between the concentration of the photosensitizers (measured in µg/mL) and the resulting count of colony-forming units (CFU/mL). (**A**) A schematic presentation of the sequential treatment of MB followed by PDZ against K. pneumoniae cells, and the observed results. (**B**) A schematic presentation of the sequential treatment of PDZ followed by MB against *K. pneumoniae* cells, and the observed results.

In the subsequent experiments, we investigated the effect of sequential administration of MB and PDZ on bacterial inactivation. Fractionated irradiation was applied with a total fluence of 30 J/cm^2^, delivered in two fractions of 15 J/cm^2^ at 20 min intervals (Figure 4). When MB (5 µg/mL) was administered before PDZ, complete inactivation of *K. pneumoniae* was achieved at PDZ concentrations of 10, 25, and 50 µg/mL (Figure 4A). In contrast, higher PDZ concentrations of 100 and 200 µg/mL attenuated the bactericidal effect of MB (5 µg/mL) by 5.2 and 3.1 log10, respectively. However, the antibacterial efficacy of higher MB concentrations (10 and 20 µg/mL) was not influenced by PDZ at any tested concentration (Figure 4A).

When the photosensitizers were applied sequentially in the order MB (5 µg/mL) → MB (5 µg/mL) → PDZ (10–200 µg/mL) → PDZ (10–200 µg/mL), with each administration followed by irradiation at 15 J/cm^2^ (total dose 60 J/cm^2^), complete bacterial inactivation was consistently observed (Figure S1A). In other words, the stepwise administration of MB followed by PDZ, with irradiation after each step, produced consistent photodynamic effects, resulting in complete bacterial killing in every replicate. A similar outcome was obtained when MB (2.5–20 µg/mL) was applied twice, followed by PDZ (25 µg/mL) applied twice under the same irradiation protocol (Figure S1B). To evaluate whether residual PS affected subsequent irradiation, bacterial suspensions were washed with PBS between treatments. In this case, the sequence MB (2.5–20 µg/mL) → PBS → PDZ (25 µg/mL) → PBS, with identical irradiation parameters, did not result in detectable inhibition of *K. pneumoniae* (Figure S1C).

When the photosensitizer sequence was reversed, with PDZ administered before MB, bacterial inactivation exhibited a linear dependence on the concentration of subsequently applied MB, with the rate of reduction inversely related to the amount of PDZ used (Figure 4B). Initial treatment with PDZ-PDT at 25 µg/mL followed by MB-PDT at 10 or 20 µg/mL achieved an 8 log10 CFU/mL reduction in *K. pneumoniae* viability (Figure 4B). In contrast, PDZ at 100 µg/mL significantly (*p* < 0.05) attenuated the antibacterial activity of MB at 5, 7.5, 10, and 20 µg/mL, while PDZ at 50 µg/mL significantly reduced the effect of MB at 5, 7.5, and 10 µg/mL (Figure 4B). Sequential administration of PDZ (25 µg/mL) → PDZ (25 µg/mL) → MB (2.5–20 µg/mL) → PDZ (2.5–20 µg/mL), each followed by irradiation at 15 J/cm^2^ (total fluence 60 J/cm^2^), consistently resulted in complete bacterial inactivation (Figure S2A). A similar outcome was obtained when PDZ at 10, 25, 50, or 100 µg/mL was applied twice, followed by two applications of MB (5 µg/mL) under the same irradiation protocol (Figure S2B). However, at a PDZ concentration of 200 µg/mL, the bactericidal activity of MB (5 µg/mL) was markedly attenuated (Figure S2B). Furthermore, when PBS washing was performed after PDI, no photooxidative effects were detected (Figure S2C), confirming that MB was the principal contributor to ROS-mediated bacterial inactivation.

### 2.3. Photobleaching of PSs, Methylene Blue (MB), Photodithazine (PDZ), and Their Mixtures

**Figure 5:**
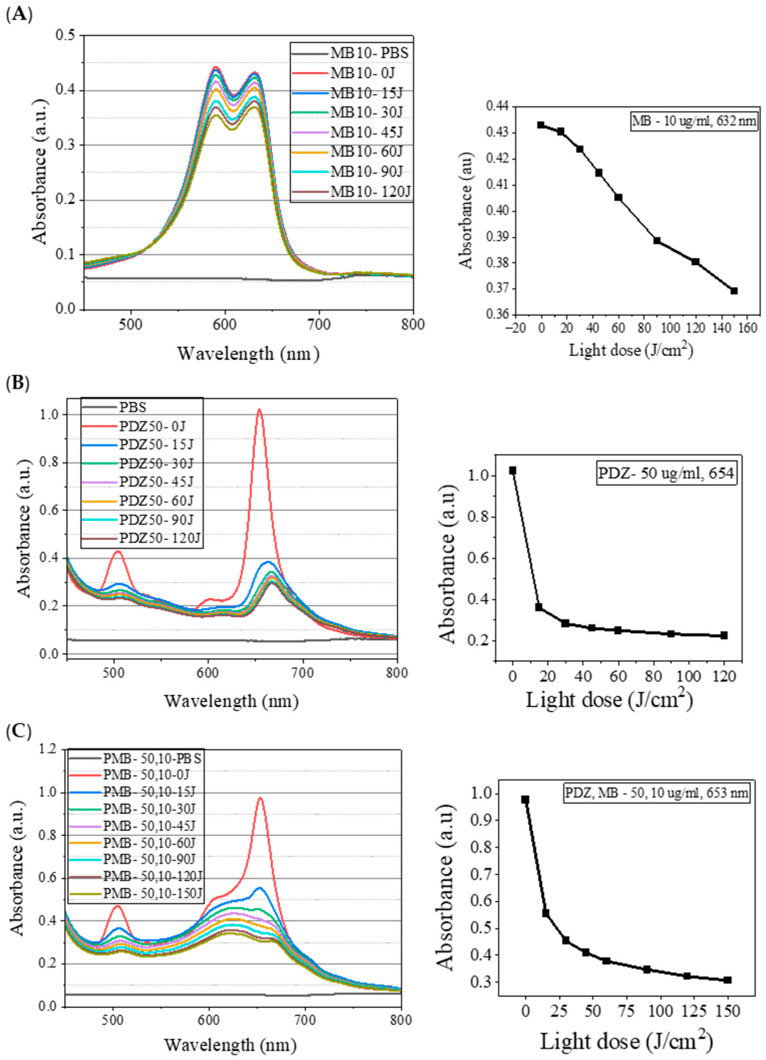
Photobleaching of photosensitizers. (**A**) Changes in the visible spectrum of Methylene Blue (MB) at a concentration of 10 µg/mL and observed variations in absorbance versus light dose (J/cm^2^) (**B**) Changes in the visible spectrum of Photodithazine (PDZ) at a concentration of 50 µg/mL and observed variations in absorbance versus light dose (J/cm^2^) (**C**) Photobleaching of mixture of MB and PDZ and observed variations in absorbance versus light dose (J/cm^2^). The irradiation was performed at 660 nm with a Biotable^®^.

We evaluated the absorption spectra of PSs, MB, PDZ, and their mixture to investigate any alterations in their photobleaching profile (Figure 5). The absorption spectra were measured in a 1 cm quartz cuvette. Major absorption bands for MB at 10 µg/mL in water were found at 590 nm and 632 nm (Figure 5A). For PDZ, absorption maxima were found at 654 nm (Figure 5B). For the mixture, the absorption bands were the summation of MB and PDZ bands together (Figure 5C). The irradiation wavelength of 660 nm was selected because both MB and PDZ exhibit strong absorption in the red spectral region, with absorption maxima that closely overlap near this wavelength. This overlap ensures efficient excitation of both photosensitizers and allows for direct comparison of their individual and combined photodynamic effects. The photobleaching results of MB, PDZ, and their mixtures are presented in Supplementary Figure S3A–C. It is worth noting that photobleaching varied significantly between MB and PDZ, where PDZ had the highest degradation rate as compared with MB (Figure 5).

## 3. Discussion

It is understood that the inactivation of microorganisms can occur in two ways: either through oxidative species formed near the microorganisms or after the microorganisms absorb the photosensitizer, leading to the formation of oxidative species. Previous studies found a synergistic effect when 6-carboxypterin (Cap) and methylene blue (MB) were combined in aPDT against *K. pneumoniae* mature biofilms. This combination was more effective than using either photosensitizer alone, significantly reducing the viability of multidrug-resistant bacteria and even enabling continued bacterial killing in the absence of light [19]. In contrast, our earlier study reported that the simultaneous use of curcumin (λ_max_ 428) and a nitrogen-based photosensitizer MB (λ_max_ 660), which absorb in different spectral regions, did not produce an additive effect against *S. aureus*, as the same level of inactivation was observed when the PSs were applied separately [20]. Our previous results implied that the combined use of MB and curcumin led to competition between them for ROS and ^1^O_2_ production because curcumin acts as an oxygen trap [20]. However, the use of different photosensitizers for bacterial photoinactivation is not widely studied, especially under optimal growth conditions. Here, we choose to investigate the aPDT effects of glucamine-salt of chlorine e6 (photodithazine, PDZ) and MB, since both are commercially available and have been used for aPDT applications.

In this study, bacteria were irradiated following a 20 min incubation period without washing out the photosensitizer (PS) mixture, as this protocol better reflects potential clinical applications [21]. In the absence of irradiation, the two PSs may interact with the substrate through different mechanisms, such as competing for binding sites on the surface of *K. pneumoniae*. Although their physical properties might be affected, such effects are expected to be minimal. In the presence of light, however, their interactions are mainly photophysical and photochemical in nature. Since both PSs absorb within overlapping spectral regions, they may compete for excitation light and subsequently for ROS generation. Depending on the concentration of PSs and the applied light dose, these interactions can lead to either synergistic or antagonistic effects due to quenching phenomena associated with photoactivation. Photodynamic therapy (aPDT) with MB alone demonstrated a significant antibacterial effect (Figure 2), consistent with previous reports. However, when MB was combined with PDZ at varying concentrations, a minimal inhibitory effect was initially observed at low PDZ (≤25 µg/mL) concentrations. Still, this activity progressively declined as the PDZ concentration increased (Figure 3). This non-linear effect suggests the presence of a threshold-like behavior that depends on multiple interacting parameters, including relative concentrations of MB and PDZ, light dose, oxygen availability, or aggregation effects that reduce MB photoexcitation, and *K. pneumoniae* density. At lower PDZ levels, MB retains sufficient availability of ROS upon illumination, leading to detectable *K. pneumoniae* inhibition. However, at higher PDZ concentrations, competitive interaction may occur, potentially altering MB’s binding sites or oxygen diffusion dynamics, thereby reducing the MB-mediated killing of *K. pneumoniae*. These findings indicate that the simultaneous use of two PSs under the same excitation wavelength results in a largely non-additive response. At elevated PDZ levels, the overall antibacterial effect tended to converge toward that observed with 15 J/cm^2^, even when the applied light dose was 30 J/cm^2^ (Figure 3). This suggests that PDZ competes with MB for photon absorption, thereby reducing the effective activation of MB. Although PDZ alone exhibited little bactericidal activity, its presence acted as a barrier that diminished MB excitation. Moreover, the reduction in efficacy may also reflect competition between PDZ molecules and bacterial targets for ROS and singlet oxygen (^1^O_2_). Accordingly, as PDZ concentration increased, *K. pneumoniae* survival also increased, with the lowest survival observed for MB in the absence of PDZ. Mechanistically, methylene blue is known to generate a higher proportion of radical species via a type I photodynamic pathway compared to singlet oxygen (type II) at the concentrations employed in this study (20 µg/mL) [22]. In contrast, PDZ (a Chlorin-e6 derivative) predominantly produces singlet oxygen rather than radical species [23]. Thus, when MB and PDZ are used together, the photodynamic reaction of MB is significantly affected by the presence of PDZ. The antagonistic effect observed here is therefore attributable to photophysical competition between the PSs, ultimately reducing MB’s efficiency.

Our results indicate that the interaction between MB and PDZ extends beyond simple light redistribution. When MB was applied first, subsequent PDZ markedly reduced bacterial killing (Figure 3A), demonstrating that MB-mediated photodynamic damage is progressive and can be interrupted. These results confirm that high PDZ concentrations interfere with MB-mediated inactivation, whereas fractionated sequential administration effectively overcomes this antagonistic effect. Potential mechanisms include competitive absorption of 660 nm light by PDZ, disruption of MB–cell interactions or quenching of MB-initiated ROS. Conversely, PDZ pretreatment moderately reduced MB efficacy (Figure 4B), highlighting the critical influence of application sequence. Kinetic factors are likely amplified by the biology of *K. pneumoniae*, whose outer membrane delays PS uptake and ROS generation, making sequential dynamics more important than instantaneous photophysical events. Molecular stoichiometry may also contribute to conditions where PDZ was present at ~3-fold excess relative to MB; competitive binding, steric hindrance, or MB aggregation could reduce MB activity. Even low PDZ concentrations suppressed MB-mediated killing, suggesting that subtle physicochemical interactions, such as altered PS localization or activation, play a role. These findings underscore that dual-PS photodynamic therapy outcomes depend critically on sequence and temporal ROS production. Future studies quantifying PS uptake and local ROS dynamics are needed to determine whether light competition or direct biochemical interactions dominate.

**Figure 6:**
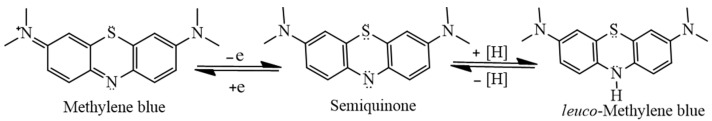
Reaction scheme for the photobleaching of methylene blue.

Singlet oxygen and ROS production are dependent on photosensitizer stability. Photobleaching causes a loss of absorption of chromophores under light exposure. Many photobleaching mechanisms can be generally classified as oxygen-dependent and independent mechanisms. Oxygen-dependent photodegradation usually involves oxidation by ^1^O_2_ and is common for porphyrinoids [24] and other chromophores such as BODIPY [25]. The attachment of electron-withdrawing groups to the ring increases its oxidation potential, resulting in greater photostability [24]. The oxygen-independent mechanism usually involves direct contact between the excited state of the PS and neighboring molecules, which can dye itself (dye-dye mechanisms) [24,26], or preferably a biological target that can be key to the target cellular response [27]. This study observed that the absorption spectrum of MB is the superposition of two bands, one with a maximum at 632 nm and the second with a maximum at 590 nm (Figure 5A). It is known that MB tends to dimerize, and therefore, the observed band at 590 nm is attributed to the dimeric form of MB [28]. It is known that chromophoric properties of MB result from its high degree of conjugation; however, it undergoes a two-step charge-transfer reaction as shown in Figure 6, which results in the formation of *leuco*-methylene, which has no significant absorbance in the visible region of the electromagnetic spectrum [29]. However, further studies are required to examine whether photodegradation is affected by oxygen or not by removing the oxygen through argon-purged solutions. Since the absorption bands in the PSs mixture were the combination of the bands of a single PS, one should keep in mind for later applications that the light source has to cover the whole range of absorption spectra for both PSs. In this study, for all concentrations evaluated, PDZ presented a high percentage of degradation (~75%), whereas MB showed a lower degradation (~15%) (Figure 5). This may be due to the shielding effect (steric hindrance) of MB molecules by PDZ, in such a way as to decrease the bleaching of MB. Regarding the mechanism of MB and PDZ, both PSs act according to the PDT mechanism (Jablonski Diagram), as reported in the literature. However, in this photodynamic protocol, two PSs were used, and both molecules act and interfere with the result of photoinactivation, stability, and photobleaching. In other words, when a PS is excited with an appropriate wavelength followed by oxidative species formation, another PS molecule can act by capturing ROS (causing degradation itself and/or protecting the other PS), as well as capturing ROS and decreasing antimicrobial efficiency.

Regarding the photodegradation of PSs under irradiation, the process generally involves five key components: light, the photosensitizers MB and PDZ, molecular oxygen in its triplet state (^3^O_2_), and the ROS generated. The mechanism can be represented by the following equations. Equation (1a,b) describe the rate of ROS formation upon irradiation of MB and PDZ (both excited at 660 nm), respectively.

(1a) d [ROS]dt = AMB [O23] [MB] − B1 [ROS] [MB]− C1 [ROS] [CPDZ]

(1b) d [ROS]dt = APDZ [O23] [CPDZ] − B2 [ROS] [MB]− C2 [ROS] [CPDZ]

In these equations, t denotes time; ROS refers to reactive oxygen species (including ^1^O_2_, O_2_^− ●^, H_2_O_2_, and ^●^OH); A_MB_ represents the light-dose coefficient (J/cm^2^) for MB irradiated at 660 nm; [^3^O_2_] is the concentration of triplet oxygen; B_1_ is the constant describing MB photobleaching at 660 nm; [MB] is the concentration of MB; C_1_ is the coefficient of PDZ photobleaching at 660 nm; [CPDZ] is the concentration of PDZ; A_PDZ_ is the light-dose coefficient (J/cm^2^) for PDZ at 660 nm; B_2_ is the constant for MB photobleaching at 660 nm; and C_2_ is the coefficient for PDZ photobleaching at 660 nm. Under these conditions, ROS production is proportional to the concentration of MB or PDZ, the availability of triplet oxygen, and the applied light dose (A_MB_ or A_PDZ_). The net ROS balance is determined by the difference between the amount generated and the fraction quenched through interactions with MB and PDZ. Equation (2a,b) describe the photobleaching rates of MB and PDZ, respectively. Photodegradation of these PSs arises from their ability to capture ROS and undergo oxidative modification of their molecular structures. The extent of this degradation depends primarily on ROS concentration and the photosensitizer levels present.

(2a) $$\frac{d\;[\mathrm{MB}]}{dt}\;=\;-\;B_{\mathrm{1or2}}\;[\mathrm{ROS}]\;[\mathrm{MB}]$$

(2b) $$\frac{d\;[\mathrm{CPDZ}]}{dt}\;=\;-\;C_{\mathrm{1or2}}\;[\mathrm{ROS}]\;[\mathrm{CPDZ}]$$

The results and corresponding equations indicate that the availability of ROS to drive oxidation reactions in target bacteria depends on the balance between their formation rates, generated through photoactivation of MB and PDZ, and their depletion via photobleaching of these PSs. This interplay establishes a competitive effect that leads to significant variation when both are present, even under the excitation of only one PS. This study has certain limitations, including the use of only a single bacterial strain, the lack of testing against other strains, and the evaluation being restricted to two individual photosensitizers and their mixture.

## 4. Materials and Methods

The experimental procedure consists of culturing microorganisms followed by a stepwise application of a photosensitizer and exposure to light. Final observations are conducted after a specified incubation period to assess the outcomes of these treatments.

### 4.1. Culture Media

All media and dilutions were prepared following clinical antimicrobial susceptibility testing guidelines. Cation-adjusted Muller-Hinton broth (CAMHB) and Muller-Hinton agar (MHA) were purchased from Hardy Diagnostics (Santa Maria, CA, USA). Brain heart infusion broth (BHIB) and brain heart infusion agar (BHIA) were purchased from Millipore (Darmstadt, Germany) and prepared according to the manufacturer’s instructions.

### 4.2. Bacterial Strain and Culture Conditions

The model strain used in this study was *Klebsiella pneumoniae* subsp. *pneumoniae*, obtained from the American Type Culture Collection (ATCC^®^ 13883™). It is cultured at 37 °C for 18 h in brain heart infusion (BHI, Millipore, Darmstadt, Germany) broth, and aliquots are frozen in BHI-glycerol at −80 °C until use. A pre-inoculum solution was prepared by mixing 1 mL of a cryotube bacteria sample with 9 mL of BHI (pH 7.4) broth, then incubated at 37 °C for 18 h in a 5% CO_2_ atmosphere on a shaker incubator at 200 rpm (MaxQ 6000, Thermo Fisher Scientific, Waltham, MA, USA). Subsequently, the cells were collected, centrifuged for 5 min at 8000 rpm, and resuspended in phosphate-buffered saline (PBS). The optical density of *K. pneumoniae* suspensions was adjusted to 0.2–0.3 at 600 nm (OD 600, Agilent Cary 60 UV-Vis, Agilent Technologies Inc., Santa Clara, CA, USA), corresponding to approximately 10^7^–10^8^ colony-forming units per milliliter (CFU/mL).

### 4.3. Photosensitizer

In our study, we used two photosensitizers (PSs) and their combinations. The photosensitizer methylene blue (MB) was acquired from Sigma-Aldrich (St. Louis, MO, USA) (USP Reference Standard, cat# 1428008). A stock solution of MB (50 µg/mL) was prepared using sterile water and stored at 4 °C in the dark. Photodithazine^®^ (PDZ), a chlorine e-6 compound, was supplied through the collaborative efforts of the University of São Paulo and Texas A&M University. Stock solutions of PDZ were prepared at a concentration of 400 µg/mL in distilled water and subsequently diluted in distilled water to achieve the desired concentrations. The mixtures of MB and PDZ solutions were obtained through dilutions from the stock solutions using PBS as a solvent. Photosensitizer solutions were prepared in microtubes wrapped in aluminum foil to safeguard against light exposure during the experiment. All irradiations were carried out under an aluminum sheet covering to prevent exposure to room light.

### 4.4. Light Source and Photodynamic Inactivation Procedure

The light source used for PDT experiments was acquired from PineTek (PINETEK LLC, 511 University Dr., College Station, TX, USA) and assembled by the Technical Support laboratory at the Physics Institute of São Carlos (USP/SP/Brazil). The device consists of a plate holder with 24 LEDs arranged in a 4 × 6 grid, providing an irradiation of 75 mW/cm^2^ and emitting evenly across the surface at 660 nm. The distance between the LED light guide and the sample surface was 80 mm. Cells were irradiated for 3 min 20 s or 6 min 40 s, corresponding to total light doses of 15 or 30 J/cm^2^ [total light dose (J/cm^2^) = fluence rate (W/cm^2^) × treatment time (s)]. The device allows control of intensity and exposure time to achieve the desired dose delivery. The power density of the incident radiation was measured using a power meter, and variations between wells stay within 10%.

The Photodynamic Therapy (PDT) procedure was established following our recent study [30]. We established three control groups: a general control group of bacteria, the control samples for the dark toxicity were only exposed to the PS (final concentrations of MB and PDZ were 20 and 200 µg/mL, respectively) without any illumination, and a light control group with bacteria exposed to light. Additionally, we included a treatment group for PDT, consisting of bacteria treated with PS and exposed to light. For the PDT and dark control groups, bacteria were incubated with different concentrations of PS for 20 min. Afterward, both the PDT and light control groups were exposed to irradiation at approximately 15, 30, or 60 J/cm^2^ using a 660 nm LED device (Biotable, LAT, USP, Brazil) [30]. Following light exposure, samples were plated on Petri dishes to count the surviving colony-forming units per milliliter (CFU/mL). For this, 10 µL of each sample was transferred into microtubes containing 90 µL of PBS to carry out the serial dilution. Aliquots of 10 µL of all dilutions of samples were uniformly spread to Petri dishes with Muller-Hinton agar in duplicate. Plates were maintained at 37 °C for 18 h to carry out the counts of CFU.

Photodynamic inactivation (PDI) experiments against *K. pneumoniae* were conducted using a full factorial design (FFD), considering two factors at two levels with one central point. All tests were performed in duplicate or triplicate, resulting in 18 experimental runs, each carried out with replicates. The independent variables were the concentration of MB (*X*_1_, µg/mL) and the concentration of PDZ (*X*_2_, µg/mL). Both were treated as continuous variables with coded levels of −1, 0, and +1, corresponding to concentrations defined from preliminary screening assays to establish suitable working ranges. The objective of this study was to investigate whether factor *X*_2_ (PDZ concentration) enhanced the antibacterial activity of MB against *K. pneumoniae*. Combined effects were evaluated using a synergistic factor (*k*), calculated according to the equation *k* = (*Y* − *Y*_X1_)/(*Y*_X1_ + *Y*_X2_). Where *k* values > 0 indicate cooperative (synergistic) effects, while *k* values < 0 indicate competitive (antagonistic) effects. Here, *Y* represents the observed log-reduction obtained for the combined treatment (average of equivalent runs), *Y*_X1_ denotes the log-reduction observed with MB under red light irradiation (660 nm), and *Y*_X2_ represents the corresponding effect of PDZ under the same conditions.

### 4.5. Photobleaching Evaluation

Absorption spectra of PSs, MB, and PDZ were measured using an Agilent Cary 60 UV-Vis spectrophotometer (OD 600, Agilent Technologies Inc., USA). Freshly prepared solutions of MB and PDZ were used for photobleaching experiments. Photobleaching experiments were conducted using a Biotable^®^ apparatus (at 660 nm). An aliquot from the medium containing the MB, PDZ, and a mixture of MB and PDZ solutions was taken at specific times (before and after irradiation) and measured using UV-visible spectroscopy in the 200–800 nm range with a quartz cuvette. The photobleaching percentage was determined by the decrease in the absorbance peak at approximately 660 nm for MB and PDZ, as both have their maximum absorbance at 660 nm.

### 4.6. Statistical Analysis

Each experiment was conducted at least three times. All primary data are shown as means with the standard deviation. The Shapiro–Wilk normality test was used to assess whether the data followed a normal distribution. Intergroup differences were analyzed using a one-way ANOVA followed by Tukey’s multiple comparison test. A *p*-value of less than 0.05 was considered statistically significant.

## 5. Conclusions

In summary, this study demonstrated that methylene blue (MB)-mediated antimicrobial photodynamic therapy (aPDT) achieved superior antibacterial activity against *Klebsiella pneumoniae*, whereas Photodithazine (PDZ) exhibited only modest effects. The simultaneous use of MB and PDZ under 660 nm irradiation did not enhance photodynamic efficacy, and sequential administration revealed clear antagonism. Notably, the addition of PDZ following MB markedly attenuated bacterial killing, indicating that MB-induced photodamage is progressive yet susceptible to interruption. Increasing PDZ concentrations further promoted bacterial survival, with the greatest inactivation consistently observed for MB alone. Pretreatment with PDZ also moderately diminished MB activity. These outcomes can be attributed to competition for reactive oxygen species (ROS), accelerated photobleaching, light attenuation, steric hindrance restricting ROS access, and the role of PDZ as an oxygen/ROS sink that preferentially quenched singlet oxygen, exacerbated its own degradation, and reduced MB photobleaching and efficacy. Collectively, these findings indicate that combining MB and PDZ provides no therapeutic advantage over MB alone under the tested conditions. While limited to in vitro assays, the results underscore the critical importance of photosensitizer selection and dosing strategies in the development of effective PDT approaches against multidrug-resistant pathogens.

## Data Availability

The original contributions presented in this study are included in the article/Supplementary Material. Further inquiries can be directed to the corresponding authors.

## Supplementary Materials

The following supporting information can be downloaded at: https://www.mdpi.com/article/10.3390/ijms262010211/s1.
