# Supplementary material for: The Combined Photosensitizers in Antimicrobial Photodynamic Therapy: The Case of Methylene Blue and Photodithazine Against Klebsiella pneumoniae
Source: Int J Mol Sci. 2025 Oct 21;26(20):10211. doi: 10.3390/ijms262010211 (PMC12562916; doi:10.3390/ijms262010211)
Supplement: Supplementary file 1 [file ijms-26-10211-s001.zip › ijms-3912495-supplementary.pdf]

Supplementary data: The combined photosensitizers in antimicrobial photodynamic therapy: the case of Methylene Blue and Photodithazine against *Klebsiella pneumoniae*

(A)

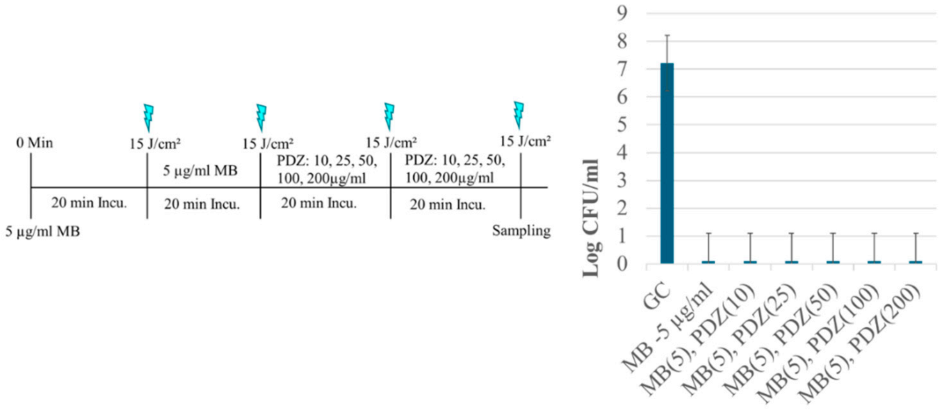

(B)

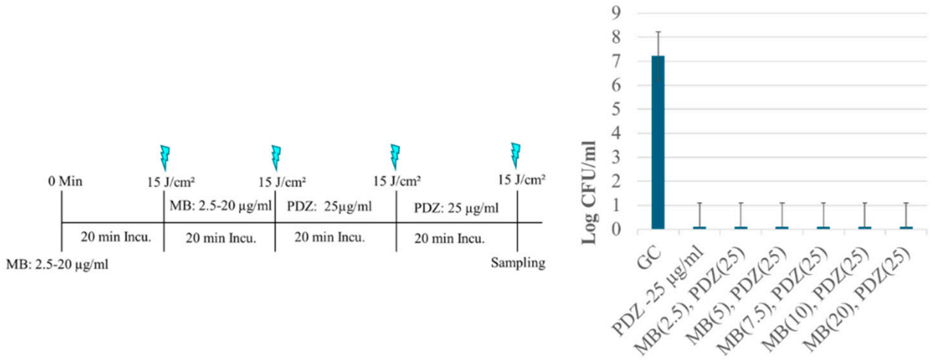

(C)

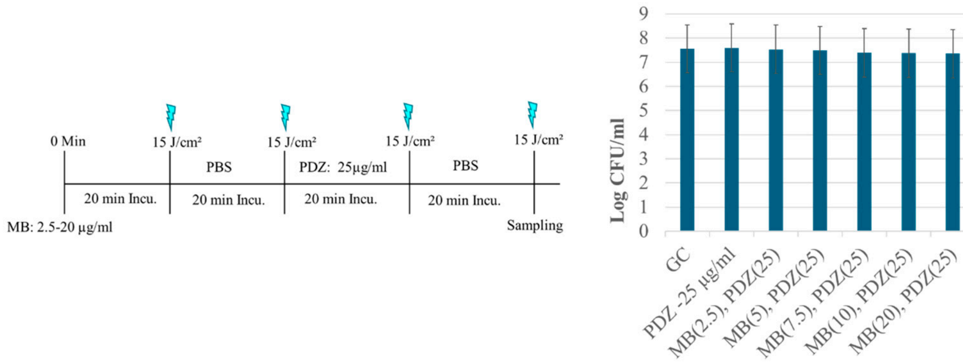

Figure S1: The schematic presentation of the sequential treatment of methylene blue (MB), followed by Photodithazine (PDZ), on the viability of *Klebsiella pneumoniae*, and the observed results. (**A**) photosensitizers were applied sequentially in the order MB (5 µg/mL) → MB (5 µg/mL) → PDZ (10–200 µg/mL) → PDZ (10–200 µg/mL), with each administration followed by irradiation at 15 J/cm^2 (total dose 60 J/cm^2). (**B**) MB (2.5–20 µg/mL) was applied twice, followed by PDZ (25 µg/mL) applied twice under the same irradiation protocol. (**C**) the sequence MB (2.5–20 µg/mL) → PBS → PDZ (25 µg/mL) → PBS, with identical irradiation parameters. The graphs illustrate the relationship between the concentration of the photosensitizers (measured in µg/mL) and the resulting count of colony-forming units (CFU/mL).

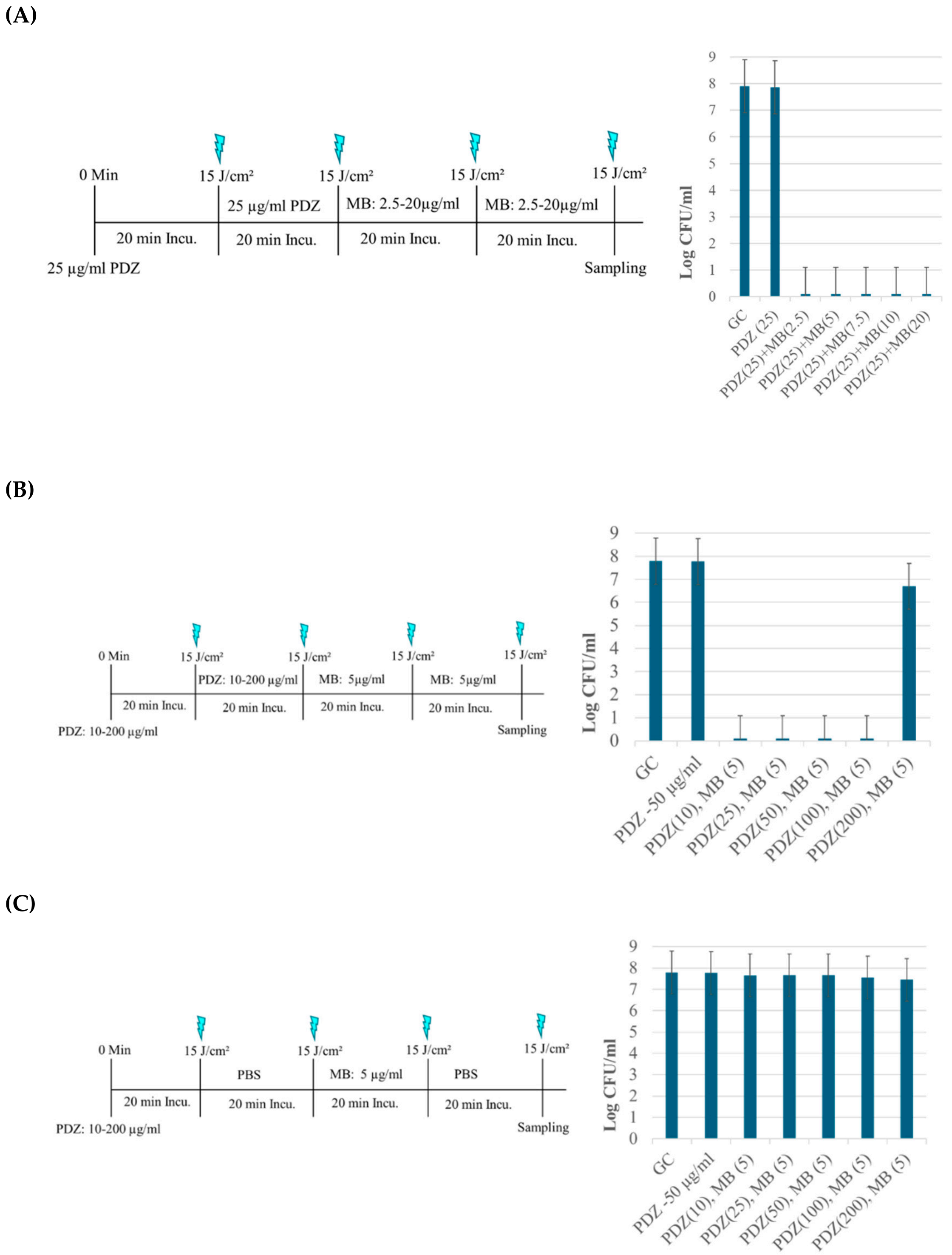

Figure S2: The schematic presentation of the sequential treatment of Photodithazine (PDZ), followed by methylene blue (MB), on the viability of *Klebsiella pneumoniae*, and the observed results. (**A**) Sequential administration of PDZ (25 µg/mL) → PDZ (25 µg/mL) → MB (2.5–20 µg/mL) → PDZ (2.5–20 µg/mL), each followed by irradiation at 15 J/cm^2 (total fluence 60 J/cm2). (**B**) PDZ at 10, 25, 50, or 100 µg/mL was applied twice, followed by two applications of MB (5 µg/mL) under the same irradiation protocol. (**C**) PBS washing was performed after PDI. The graphs illustrate the relationship between the concentration of the photosensitizers (measured in µg/mL) and the resulting count of colony-forming units (CFU/mL).

Photobleaching of photosensitizers Methylene Blue (MB) and Photodithazine (PDZ)

Photobleaching of photosensitizers Methylene Blue (MB)

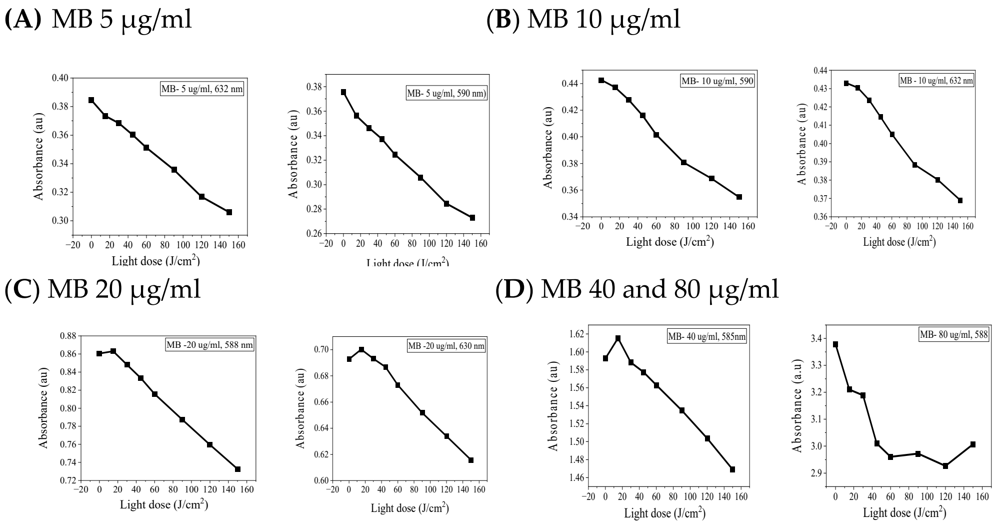

Figure S3A. Changes in the visible spectrum of Methylene Blue (MB) at a concentrations of 5 - 80 μg/mL and observed variations in absorbance versus light dose (J/cm²). The irradiation was performed at 660 nm with a Biotable®.

Photobleaching of photosensitizer Photodithazine (PDZ)

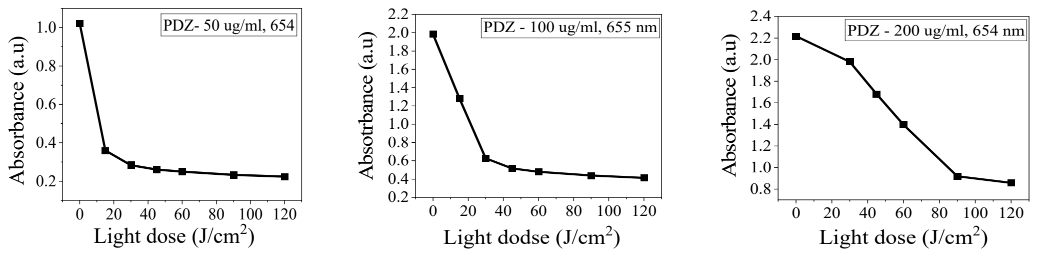

Figure S3B. Changes in the visible spectrum of Photodithazine (PDZ) at a concentration of 50, 100 and 200 μg/mL and observed variations in absorbance versus light dose (J/cm²). The irradiation was performed at 660 nm with a Biotable®.

Photobleaching of photosensitizer mixtures

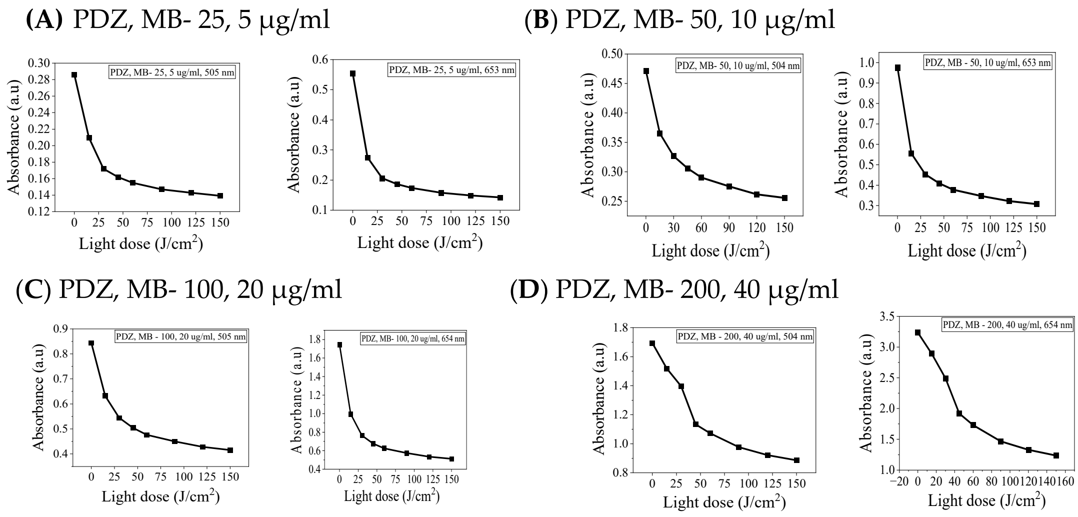

Figure S3C. Photobleaching of mixture of MB and PDZ and observed variations in absorbance versus light dose (J/cm^2). The irradiation was performed at 660 nm with a Biotable®.
